# MAVS: A Two-Sided CARD Mediating Antiviral Innate Immune Signaling and Regulating Immune Homeostasis

**DOI:** 10.3389/fmicb.2021.744348

**Published:** 2021-09-09

**Authors:** Yunqiang Chen, Yuheng Shi, Jing Wu, Nan Qi

**Affiliations:** ^1^Collaborative Innovation Center of Yangtze River Delta Region Green Pharmaceuticals, College of Pharmaceutical Sciences, Institue of Engineering Biology and Health, Zhejiang University of Technology, Hangzhou, China; ^2^Shanghai Key Laboratory of Medical Epigenetics, Institutes of Biomedical Sciences, Fudan University, Shanghai, China

**Keywords:** antiviral signal transduction, immune homeostasis, mitochondrial antiviral signaling protein, post-translational modification, viral evasion

## Abstract

Mitochondrial antiviral signaling protein (MAVS) functions as a “switch” in the immune signal transduction against most RNA viruses. Upon viral infection, MAVS forms prion-like aggregates by receiving the cytosolic RNA sensor retinoic acid-inducible gene I-activated signaling and further activates/switches on the type I interferon signaling. While under resting state, MAVS is prevented from spontaneously aggregating to switch off the signal transduction and maintain immune homeostasis. Due to the dual role in antiviral signal transduction and immune homeostasis, MAVS has emerged as the central regulation target by both viruses and hosts. Recently, researchers show increasing interest in viral evasion strategies and immune homeostasis regulations targeting MAVS, especially focusing on the post-translational modifications of MAVS, such as ubiquitination and phosphorylation. This review summarizes the regulations of MAVS in antiviral innate immune signaling transduction and immune homeostasis maintenance.

## Introduction

The battle between humans and viruses is never ending. In recent years, new emerging viruses, such as SARS-CoV-2 (also referred to as 2019-nCoV), pose a tremendous threat to public health. Our bodies developed different immune systems to defend ourselves against viral infection. Innate immunity is the body’s first line of defense against foreign pathogens. It consists of tissue barriers (skin and mucous membranes), phagocytes (macrophages and neutrophils), dendritic cells (DC), and killer cells (Thompson and Locarnini, 2007). Innate immunity produces interferons (IFNs) and pro-inflammatory factors to inhibit and eliminate the invading pathogens and maintain the hots immune homeostasis. Meanwhile, IFNs have immunomodulatory effects and can activate acquired immunity through antigen presentation. In addition, innate immunity also plays a key role in inhibiting tumor growth and metastasis.

## The Structure and Function of Rlrs and Mavs

The activation of the innate immune system requires pattern recognition receptors (PRRs) to recognize pathogen-associated molecular patterns (Kawai and Akira, 2010; Broz and Monack, 2013; Cao, 2016). retinoic acid-inducible gene I (RIG-I)-like receptors (RLRs) are the main PRRs in the cytoplasm that sense and respond to viral RNA (Hiscott et al., 2010), including RIG-I, melanoma differentiation-associated gene 5 (MDA5), and laboratory of genetics and physiology 2 (LGP2; Yoneyama et al., 2004; Katze et al., 2008), which all belong to the family of RNA helicases containing DExD/H characteristic domains. RIG-I and MDA5 contain three identical basic domains. The N-terminal caspase-recruitment domains (CARDs) are responsible for transducing signals to downstream protein factors and then activate nuclear factor κB (NF-κB) and INF regulatory factor 3/7 (IRF3/7)-related signaling pathways. A central DExD/H-box helicase domain and a C-terminal domain (CTD) can bind to the 5′ppp-RNA tail of viral RNA (Yoneyama et al., 2004; Hornung et al., 2006). RIG-I recognizes short-stranded viral RNA, while MDA5 recognizes long-stranded viral DNA with the help of LGP2. RLRs play critical roles in the antiviral innate immunity system *via* the induction of type I INF and its downstream INF-stimulated genes.

**Figure 1:**
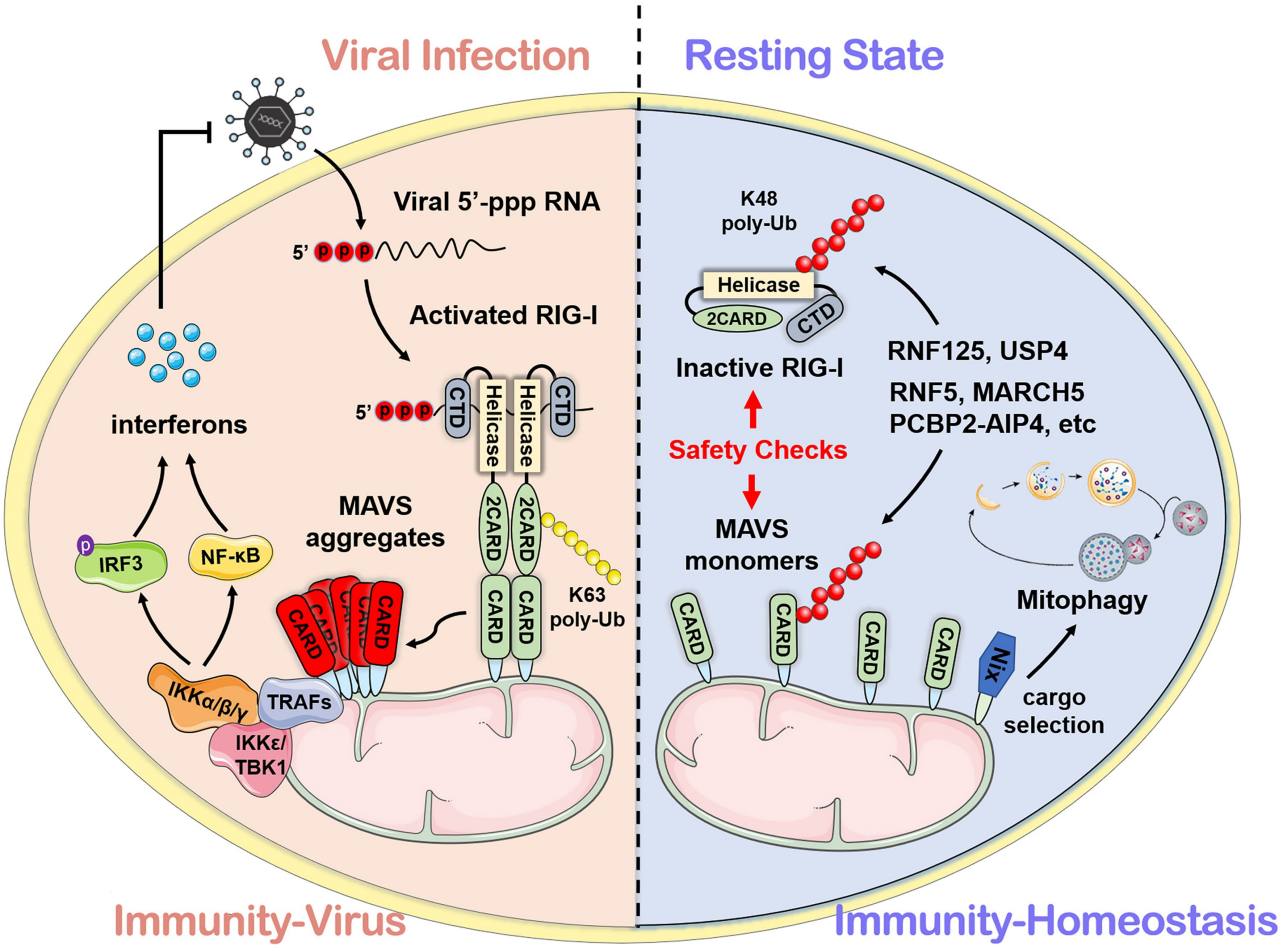
The mechanism mediated by mitochondrial antiviral signaling protein (MAVS) during viral infection and homeostasis. After recognizing viral RNA, the caspase-recruitment domain (CARD) of retinoic acid-inducible gene I interacts with the N-terminal CARD domain of MAVS and subsequently activates downstream antiviral signals. In the resting state, MAVS protein maintains immune homeostasis through self-inhibition and the ubiquitin-proteasome system.

Mitochondrial antiviral signaling protein (MAVS; also known as IPS-1/VISA/Cardif) functions as a platform for antiviral innate immune signal transduction (Kawai et al., 2005; Meylan et al., 2005; Seth et al., 2005; Xu et al., 2005). MAVS consists of 540 amino acids. The N-terminal CARD can interact with the CARD of RIG-I/MDA5. The C-terminal is the transmembrane domain (TM) which locates MAVS on the outer mitochondrial membrane. Moreover, a proline-rich region contains three active motif binds to the downstream E3 ubiquitin ligase TRAFs (Seth et al., 2005). Upon viral infection, MAVS forms prion-like aggregates by receiving the signal from the cytosolic RNA sensor RIG-I and subsequently activates downstream NF-κB and IRF3/7-related signal pathways, switching on the type I IFN signaling to produce type I IFNs (e.g., IFN-α and IFN-β) and other cytokines (e.g., TNF-α and interleukins) through a series of cascade reactions (Castanier et al., 2012; Liu et al., 2017a). Figure 1 shows the framework of this review.

The current research and exploration on the regulation mechanism of MAVS mainly focus on four aspects. The first is the regulation of the molecular activity of MAVS by protein interactions. For example, LGP2 interacts with the TM domain of MAVS to prevent MAVS from recruiting E3 ligase TRAF3. The second is the regulation of mitochondrial polymorphism on the molecular activity of MAVS. Studies have shown that factors affecting the physical state of mitochondria, such as mitochondrial fusion, changes in membrane potential, and the level of reactive oxygen species (ROS), also alter the formation of MAVS aggregates (de Brito and Scorrano, 2008; Onoguchi et al., 2010; Zhao et al., 2012). Thirdly, post-translational modification (PTM) on MAVS molecular activity has become a pivotal host antiviral innate immune signaling regulation. Lastly, cells have evolved many automated mechanisms to maintain immune homeostasis to balance the activation and suppression of the innate immune response.

## Ptms Control of Mavs

### Brief Conception of PTMs

**Figure 2:**
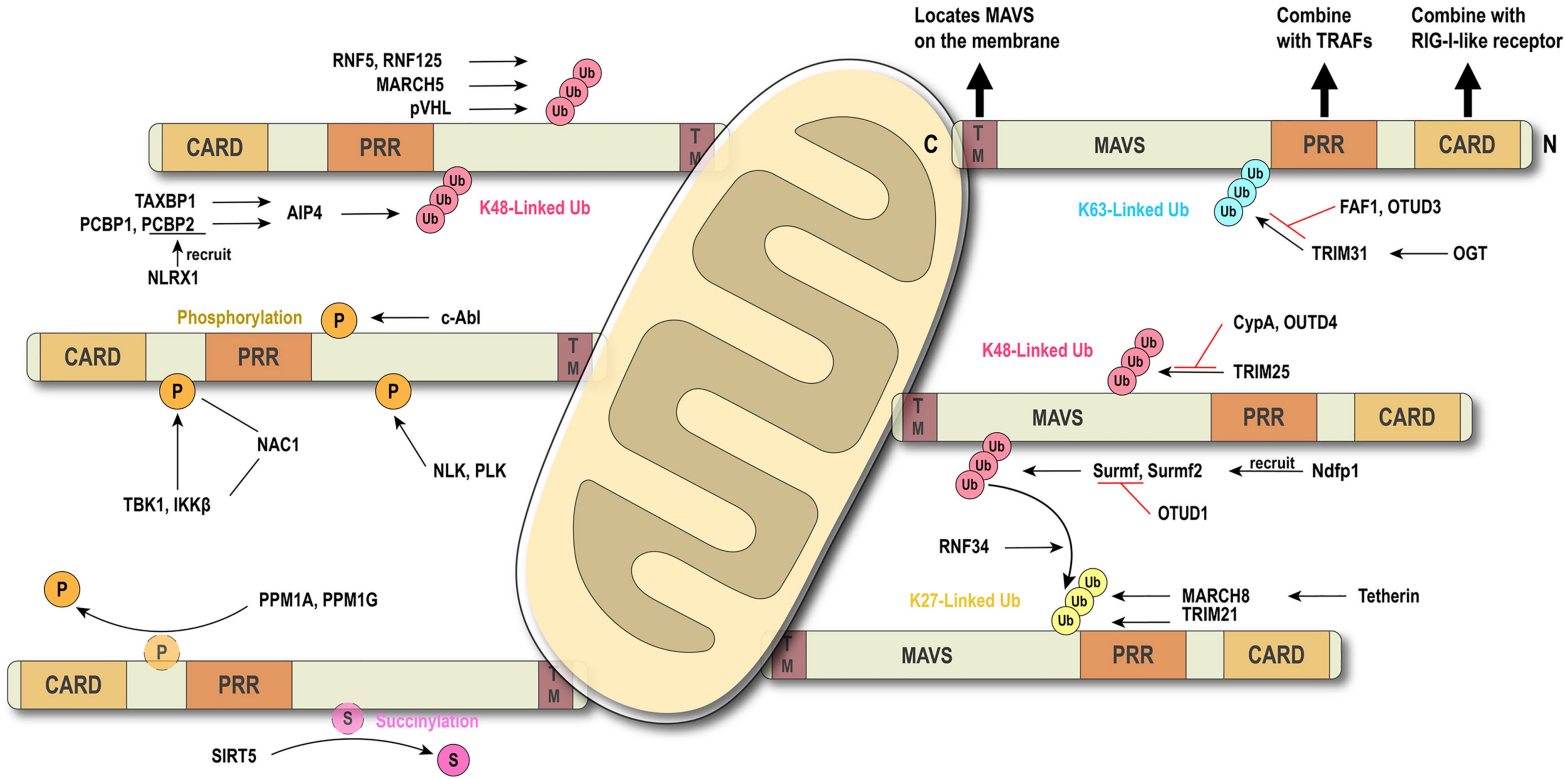
Brief conception of ubiquitination and phosphorylation in MAVS regulation. MAVS contains three key motifs and locates on the outer membrane of mitochondria. Ubiquitination and phosphorylation are the most common types of post-translational modifications (PTMs) which regulate MAVS in the innate immune signaling pathway.

Post-translational modifications of proteins can dynamically regulate the partitioning, transport, and physical interaction of pivotal molecules in the immune process (Diskin et al., 2021). It is emerging as a key mechanism by which intracellular metabolites can modulate immunity (Deribe et al., 2010; Liu et al., 2016). The precursor protein is generally inactive and often requires a series of post-translational processing to become a functional mature protein. The stability and activity of the protein are regulated by covalently binding with new functional groups, such as phosphate, methyl, and acetate. It has been demonstrated that traditional aluminum electrolytic phosphorylation and ubiquitination and non-traditional modifications, such as carbonylation and hydroxylation (Yang et al., 2017; Boeynaems and Gitler, 2018; Strowitzki et al., 2019), can target inflammatory responses related to PRRs through natural immune signaling pathways. These reversible modifications are catalyzed by specific enzymes. PTMs of MAVS are listed in Table 1, with the bested-studied phosphorylation and ubiquitination illustrated in Figure 2.

**Table 1:** The PTMs of MAVS.

Regulator	PTMs	Site	Function
TRIM31	K63-linked Ubiquitination	K10/K311/K461	Promotes the aggregation to positively regulate antiviral immunity (Liu et al., 2017a)
OGT	O-linked β-N-acetylglucosamine	S366	Positively regulates signaling by promoting the TRIM31-mediated K63-linked ubiquitination (Li et al., 2018; Song et al., 2019)
FAF1	K63-linked Ubiquitination	NA	Negatively regulates the antiviral signaling by competing with the TRIM31-mediated K63-linked polyubiquitination (Dai et al., 2018)
OTUD3	K63-linked Ubiquitination	K129	OTUD3 directly hydrolyzes the K63-linked poly-ubiquitinated MAVS (Zhang et al., 2020c)
RNF125	K48-linked ubiquitination	NA	Interacts with MAVS and initiates proteasomal degradation (Arimoto et al., 2007)
RNF5	K48-linked ubiquitination	K362/K461	Interacts with MAVS and initiates proteasomal degradation (Zhong et al., 2010)
OTUD4	K48-linked ubiquitination	NA	OTUD4 regulates the stability of MAVS (Liuyu et al., 2018)
MARCH5	K48-linked ubiquitination	K7K500	Interacts with MAVS and initiates proteasomal degradation (Yoo et al., 2015; Park et al., 2020)
pVHL	K48-linked ubiquitination	K420	Tumor suppressor pVHL targets Lys420 residue of MAVS and initiates proteasomal degradation (Du et al., 2015)
TRIM25	K48-linked Ubiquitination	K7/K10	Interacts with MAVS and initiates proteasomal degradation (Castanier et al., 2012)
CypA	K48-linked Ubiquitination	NA	Negatively regulates the antiviral signaling by competing with the TRIM25-mediated K48-linked polyubiquitination (Liu et al., 2017b)
AIP4	K48-linked Ubiquitination	K371/K420	Initiates proteasomal degradation of MAVS (You et al., 2009)
NLRX1	K48-linked Ubiquitination	NA	Recruits PCBP2 to mediate proteasomal degradation (Qin et al., 2017)
PCBP2	K48-linked Ubiquitination	NA	Recruits AIP4 to degrade MAVS (Xia et al., 2015)
PCBP1	K48-linked Ubiquitination	NA	PCBP1 and PCBP2 degrade MAVS with the same mechanism, PCBP1 is stably expressed in both viral and resting states, while the basic expression of PCBP2 overlaps, but it is rapidly induced after the virus infection (Zhou et al., 2012)
TAX1BP1	K48-linked Ubiquitination	NA	TAX1BP1 has a similar effect to PCBP1/PCBP2 (Choi et al., 2017)
Ndfp1	K48-linked Ubiquitination	NA	Recruits Smurf1 and Smurf2 to initiate proteasomal degradation of MAVS (Wang et al., 2012)
Smurf1/ Smurf2	K48-linked Ubiquitination	NA	Initiates proteasomal degradation of MAVS (Wang et al., 2012; Pan et al., 2014)
OTUD1	Removal of Smurf1 ubiquitination	NA	OTUD1 removes the ubiquitination of Smurf1 to promote the K48-linked ubiquitination of MAVS (Heise et al., 2018)
TRIM21	K27-linked Ubiquitination	K325	TRIM21 catalyzes the K27-linked ubiquitination to promote the recruitment of TBK1
MARCH8	K27-linked Ubiquitination	K7	Induces MAVS lysosomal autophagy with the help of NDP52 (Jin et al., 2017; Jin and Cui, 2018)
RNF34	K27-linked Ubiquitination	K311	Promotes the autophagy degradation of MAVS aggregates (He et al., 2019)
c-Abl	Phosphorylation	Y9/Y30/Y71	c-Abl positively regulates RLR signaling by phosphorylating MAVS (Cheng et al., 2016)
TBK1 and IKKβ	Phosphorylation	S442	TBK1 and IKKbβ directly phosphorylate MAVS and recruit IRF3 for its phosphorylation by TBK1 (Liu et al., 2015)
NAC1	Phosphorylation	NA	NAC1 takes bridge effect between MAVS and TBK1 (Xia et al., 2019)
Nemo-like kinase (NLK)	Phosphorylation	S121/S212/S258/S329	NLKs phosphorylate and degrade MAVS by associating with MAVS (Li et al., 2019a)
PPM1A(PPMCα)/PPM1G	Dephosphorylation	NA	PPM1A directly dephosphorylates the phosphorylated MAVS induced by TBK1
PLK1	Phosphorylation	T234	PLK1 phosphorylates the central T234 residue of MAVS (Vitour et al., 2009)
Sirtuin 5 (SIRT5)	Succinylation	K7	SIRT5 desuccinylate MAVS at K7 to diminish MAVS aggregation (Liu et al., 2020)

### MAVS Regulation by Ubiquitination

Ubiquitination is a common PTM in the RLR signaling pathway. E3 ubiquitin ligase TRIM31-mediated K63-linked ubiquitination plays a positive role in the antiviral immune pathway. Upon viral infection, TRIM31 is recruited to MAVS and catalyze the K63-linked polyubiquitination at K10, K311, and K461, leading to the higher efficient formation of MAVS prion-like aggregates (Liu et al., 2017a). The O-GlcNAcylation of MAVS on S366 mediated by OGT promotes TRIM31-mediated ubiquitination, thereby facilitating the activation of IRF3 and the production of IFN-β. This discovery clarifies the important role of the glucose metabolism pathway in antiviral immunity (Li et al., 2018; Song et al., 2019). Conversely, scaffold protein FAF1 negatively regulates the antiviral signal by competing with the TRIM31-mediated K63-linked polyubiquitination and diminishing the prion-like aggregation of MAVS. After viral infection, IKKε mediates FAF1 phosphorylation and, following acetylation, degradation and consequent enhancement of the MAVS antiviral signaling (Dai et al., 2018). As an acetylation-dependent deubiquitinase, OTUD3 can restrict IFN signaling by directly hydrolyzing the K63-linked poly-ubiquitinated MAVS at K129 residue (Zhang et al., 2020c).

Several lines of evidence suggest that K48-linked ubiquitination catalyzed by different E3 ligases is also critical in antiviral innate immunity (Zhong et al., 2010; Du et al., 2015; Yoo et al., 2015; Liuyu et al., 2018; Park et al., 2020). Compared with the K63-linked ubiquitination that positively regulates MAVS aggregation, TRIM25-mediated K48-linked ubiquitination negatively regulates the RLR signaling pathway through MAVS degradation by the proteasome (Castanier et al., 2012). However, this phenomenon can be competitively suppressed by CypA (Liu et al., 2017b). It has been reported that nucleotide-binding oligomerization domain-like receptor X1 (NLRX1) inhibits the production of IFN (Qin et al., 2017), and poly(rC) binding protein 2 (PCBP2) recruits the E3 ligase AIP4 (also known as ITCH) containing the HECT domain to assist NLRX1 initiating MAVS ubiquitination and degradation (You et al., 2009; Xia et al., 2015). Further research shows that PCBP1 and PCBP2 degrade MAVS with the same mechanism. The difference is that PCBP1 is stably expressed both in viral and resting states, while the basic expression of PCBP2 overlaps, but it is rapidly induced after the virus infection (Zhou et al., 2012). At the same time, TAX1BP1 has also been reported to have a similar effect to PCBP1 and PCBP2 (Choi et al., 2017). Smurf1 and Smurf2 are recruited by Ndfp1, a member of the NEDD4 family, and also mediate the K48-linked ubiquitination and degradation of MAVS (Wang et al., 2012; Pan et al., 2014), whereas OTUD1 inhibits RLR signaling by removing the Smurf1-mediated ubiquitination (Heise et al., 2018). Membrane-associated RING-cysteine-histidine (MARCH) proteins also can directly or indirectly regulate the ubiquitination of MAVS (Zheng, 2021). MARCH5 mediates K48-linked ubiquitination and induces the proteasomal degradation of MAVS by transferring the ubiquitin to K7 and K500 of MAVS (Yoo et al., 2015).

Besides K63-linked and K48-linked ubiquitination, TRIM21 can catalyze the K27-linked ubiquitination to promote the recruitment of TBK1 through the interaction with MAVS, positively regulating the antiviral signaling (Xue et al., 2018). After viral infection, E3 ubiquitin ligase MARCH8 is recruited by antiviral factor Tetherin (BST2/CD317) and mediates K27-linked ubiquitination of MAVS at K7. NDP52 induces proteasomal degradation after recognizing the K27-linked ubiquitination signal (Jin et al., 2017), and NDP52 can also trigger CALCOCO2-directed autophagic degradation (Jin and Cui, 2018). NDP52 is the bridge that connects the ubiquitinated protein and the autophagy-mediated component. Another study has reported that NDP52 recognizes the K63-to-K27 linked ubiquitination transition signal mediated by RNF34 on the K311 of MAVS, efficiently promoting the autophagy degradation of MAVS aggregates (He et al., 2019).

### MAVS Regulation by Phosphorylation

Phosphorylation and dephosphorylation have been reported to play a critical role in antiviral innate immunity. c-Abl positively regulates RLR signaling by phosphorylating MAVS at Y9, Y30, and Y71 (Cheng et al., 2016). After phosphorylation by the kinases TBK1 and IKKs at residue S442, pMAVS binds to IRF3, thereby recruiting IRF3, which is phosphorylated and activated by TBK1 to activate antiviral response (Liu et al., 2015). Xia et al. have identified NAC1, a BTB/POZ family member, as a bridge between MAVS and TBK1 (Xia et al., 2019). Conversely, dephosphorylation functions as a switch to turn off antiviral signaling. PPM1A (also known as PP2Cα), a phosphatase, directly dephosphorylates the phosphorylated MAVS induced by TBK1 and subsequently silences the cytoplasmic RNA sensor signal. Similar to PPM1A, PPM1G also dephosphorylates MAVS (Xiang et al., 2016). A Nemo-like kinase is reported to phosphorylate and induce MAVS degradation by associating with MAVS at 451 to 500 of the C-terminal region (Li et al., 2019a). Polo-like kinase PLK1 phosphorylates the central T234 residue of MAVS, while polo-box domain of the PLK1 phospho-independently associates the C terminus of MAVS to destroy the combination of MAVS and downstream partners like TRAF3 (Vitour et al., 2009).

Protein succinylation caused by succinyl-CoA is a newly discovered novel PTM (Yang and Gibson, 2019). Research shows that MAVS is succinylated under viral stimulation. Sirtuin 5 desuccinylates MAVS at K7 to diminish and antagonize the MAVS aggregation and antiviral response (Liu et al., 2020). Although the phosphorylation and ubiquitination of MAVS have been extensively studied, further investigations are still needed to discover new regulators and mechanisms.

## Virus Immune Evasion Against Rig-I-Mavs

**Figure 3:**
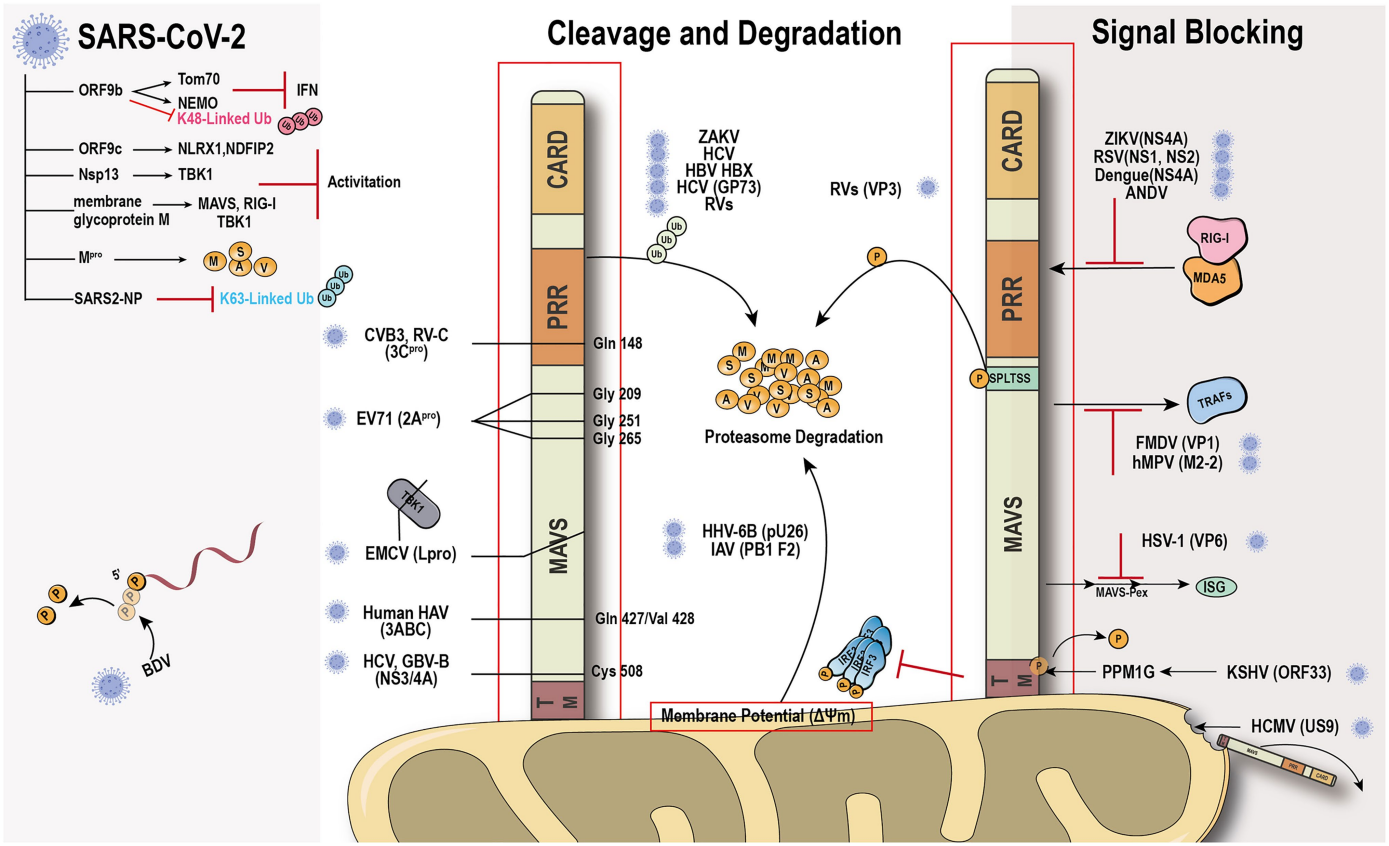
Viral immune evasion mechanism against MAVS. Viruses have evolved multiple immune evasion mechanisms targeting and sequestering MAVS from the signaling pathway directly.

The global outbreak of COVID-19 raised the scientific interest in how the virus escapes the host rigorous immune system to survive and duplicate. A study found that the Borna disease virus (BDV) genome trimming turns triphosphate end into monophosphate, effectively preventing recognition by RIG-I (Schneider et al., 2007). Compelling evidence shows that the virus has evolved various mechanisms to antagonize the innate immune response (Figure 3). The virus immune evasion methods in the RIG-I-MAVS signaling pathway can be roughly divided into two categories.

Viral protein directly cleaves or degrades MAVS to avoid the activation of immune signal transduction. Hepatitis C virus (HCV) and GB virus B serine protease NS3/4A can dislodge the N-terminal fragment of MAVS from the mitochondria and endogenous cleavage of MAVS at Cys508 (Chen et al., 2007; Xu et al., 2020). Encephalomyocarditis virus (EMCV) lead protease (L^pro^) cleaves the RLR signaling proteins MAVS and TBK1 (Jackson et al., 2020). Coxsackievirus B3 and Rhinovirus C cleaves MAVS at Gln-148 occurred within its proline-rich region (Pang et al., 2017). Enterovirus 71 proteinase 2A^pro^ cleaves MAVS at Gly209, Gly251, and Gly265, between the proline-rich and transmembrane domains (Coyne et al., 2013). 2A^pro^ has the same function as 3Cpro (Feng et al., 2014). The difference is that 2Apro cleaves MDA5 and MAVS, while 3Cpro cleaves RIG-I and MAVS. The cleavage site of viral protease is different depending on the species. HAV 3ABC protease derived from bats cleaves MAVS at Glu463/Gly464, while the protease from human cleaves MAVS at Gln427/Val428 (Feng et al., 2019). Besides direct cleavage, some viruses, such as ZIKV and HCV, can mediate MAVS degradation through ubiquitin-proteasome systems (Wei et al., 2010; Li et al., 2019b). Viral factor pU26 of human herpesvirus 6B can affect the mitochondrial membrane potential and target MAVS for degradation. The rotaviruses (RVs) RNA methyl- and guanylyl transferase (VP3) proteasomal degrade MAVS by the phosphorylation of SPLTSS motif (Ding et al., 2018b). In addition to VP3 of RVs, VP2 of EMCV and the Golgi protein 73 of HCV can interact with MAVS to induce proteasomal degradation (Zhang et al., 2017; Han et al., 2021). PB1-F2 protein of avian influenza A (H7N9) viruses inhibits MAVS aggregation, resulting in the accumulation and degradation of unaggregated MAVS on the mitochondrial membrane (Cheung et al., 2020).

Another “smart” strategy for viral evasion is to sequester MAVS and thus block the antiviral signal transduction through the RIG-I-MAVS pathway. Many non-structural proteins of the virus, such as NS1 and NS2 of respiratory syncytial virus (Ling et al., 2009), NS4A of dengue virus (He et al., 2016), a non-structural protein of unknown function from Andes orthohantavirus (Vera-Otarola et al., 2020), and NS1, NS2B/3, NS4A, NS4B, and NS5, have been shown to block the MAVS signaling transduction platform to inhibit INF production (Wu et al., 2017; Ma et al., 2018; Ding et al., 2018a; Hu et al., 2019; Lundberg et al., 2019; Schilling et al., 2020). The ORF33 of Kaposi’s sarcoma-associated herpesvirus binds to the TM domain of MAVS through the dephosphatase PPM1G, not only inhibiting the phosphorylation of MAVS but also dephosphorylating MAVS to prevent IRF3 recruitment (Yu et al., 2020). Human cytomegalovirus glycoprotein US9 induces the leakage of MAVS into the cytoplasm/ER by disrupting the potential and integrity of the mitochondrial membrane (Choi et al., 2018). Herpes simplex virus 1 tegument protein VP16 dampens the MAVS-Pex signaling and the expression of the immediate-early ISGs (Zheng and Su, 2017). Moreover, tegument protein VP1 of foot-and-mouth disease virus and M2-2 protein of human metapneumovirus block the specific recruitment of TARFs to MAVS (Mossman et al., 2013; Ekanayaka et al., 2020).

SARS-CoV-2 has caused worldwide financial loss and social disruption. Like other positive-sense RNA viruses, SARS-CoV-2 and its counterpart SARS-CoV employ various viral proteins to escape host immune surveillance (Gatti et al., 2020). The accessory protein ORF9b from SARS-CoV has been identified as an IFN antagonist that targets MAVS and promotes its proteasomal degradation, whereas SARS-CoV-2 ORF9b interacts with another mitochondrial protein TOM70 to interfere with the IFN signaling (Shi et al., 2014; Jiang et al., 2020; Kreimendahl and Rassow, 2020; Gordon et al., 2020a,b). Our recent evidence shows that SARS-CoV-2 ORF9b can also target the NF-κB essential modulator NEMO upon viral infection and inhibits its K63-linked ubiquitination, thus disrupting the canonical IKKα/β/γ-NF-κB signaling and subsequent IFN production (Wu et al., 2021). Further study found that MAVS activation can be regulated through the interaction of ORF9c and MAVS signal negative regulators (NLRX1, NDFIP2) or Nsp13 and MAVS effector TBK1 (Gordon et al., 2020b). The membrane glycoprotein M inhibits multiprotein complex formation by interacting with RIG-I, MAVS, and TBK1, and its TM1/2 domains are essential for this inhibitory effect (Zheng et al., 2020; Fu et al., 2021). Another study showed that the Mpro shows similar proteasome functionality as the main protease of SARS-CoV-2 (Wu et al., 2020). Recently, a study identified that SARS-CoV-2 nucleocapsid protein induces innate immune evasion by inhibiting K63-linked poly-ubiquitination and aggregation of MAVS (Wang et al., 2021). These works indicate MAVS plays an important role in the host against SARS-Cov-2 infection.

Although many proteins synthesized by viruses to suppress host antiviral activity have been recognized, ways for viruses to evade the surveillance of the innate immune system remain largely unknown. Recently, a study demonstrated that the Hepatitis B virus activates glycolysis and promotes lactate binding to MAVS to prevent aggregation and mitochondrial localization (Zhou et al., 2021). Another article reported that lactate is a natural suppressor of RLR signaling targeting MAVS (Zhang et al., 2019). These data indicated that a new field of virus immune evade mechanism may have been discovered.

## Mavs Immune Homeostasis Maintenance

**Figure 4:**
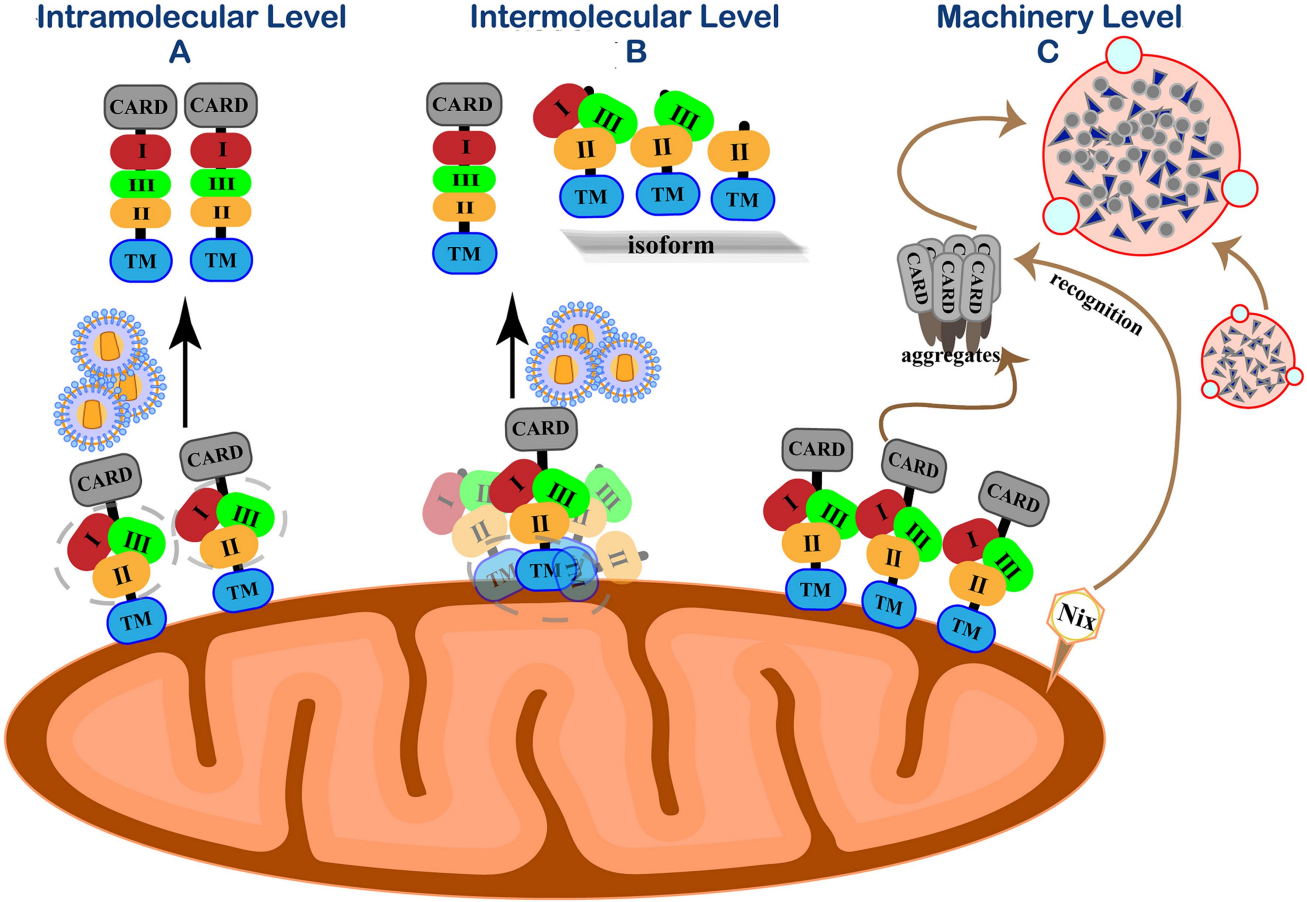
Self-inhibition mechanism of MAVS in resting state. **(A)** Intramolecular level. Three functional areas of MAVS are wrapped by its adjacent regions to maintain immune homeostasis. **(B)** Intermolecular level. N-terminally truncated isoforms interact with MAVS by their TM domain homotypic interactions to spatially isolating MAVS monomer molecules from each other on the outer mitochondrial membrane. **(C)** Machinery level. Eliminate accumulated MAVS through Nix-mediated selective autophagy.

RIG-I is wrapped by the CTD and maintains a “self-inhibiting” spatial conformation in the resting state. When RIG-I detects a foreign invading RNA virus, its CTD specifically binds to RNA molecular signals to change the spatial conception to release self-inhibition conformation and activates the antiviral immune signaling pathway. Accumulating evidence indicates that the phosphorylation of IRF3 and the production of IFN-β under viral stimulation need MAVS form prion-like aggregates under viral stimulation (Hou et al., 2011). However, MAVS will spontaneously accumulate in its natural state to form active aggregates without stimulating foreign viruses or other factors and activate downstream signaling pathways. The imbalance of antiviral immune response may lead to many autoimmune diseases, such as systemic lupus erythematosus and psoriasis (Pothlichet et al., 2011; Buskiewicz et al., 2016; Zhang et al., 2016). Therefore, in addition to the negative post-translational regulation of MAVS after the viral infection, in the resting state, the cell must have a strict regulation mechanism to prevent the spontaneous aggregation of MAVS and maintain its monomeric form, thereby ensuring the “closed” state of the RIG-I-MAVS signaling pathway (Figure 4).

PCBP1 has a certain regulatory effect on MAVS, and it persists in resting cells to prevent MAVS from accumulating (Zhou et al., 2012). RNF115 is related to the MAVS protein regulation in resting cells. RNF115 can continuously catalyze the K48-linked ubiquitination of MAVS and regulate the steady-state MAVS level in uninfected cells (Zhang et al., 2020b). Shi et al. revealed a self-inhibition mechanism that regulates the activity of MAVS in unstimulated cells (Shi et al., 2015). They identified three MAVS active regions with different functions, a region consisting of 401–450 amino acids (region III) for TBK1/IRF3 activation and two MAVS regions responsible for IKK/NF-κB activation (region I aa-138-152 and area II aa-451-465). Similar to the self-inhibition of RIG-I, the three functional areas of MAVS are wrapped by its adjacent regions to avoid the auto-activation of MAVS.

The polycistronic transcript of human MAVS generates a full-length MAVS from ORF1. Unlike full-length MAVS, its ORF2 can generate an N-terminal 141-amino acid truncated isoform that exerts dominant-negative effects on MAVS aggregation and its activity (Brubaker et al., 2014; Minassian et al., 2015). N-terminally truncated isoforms cannot form aggregates by themselves as the N-terminal CARD domain by which MAVS aggregation is mediated. Instead, these isoforms interact with full-length MAVS by their TM domain homotypic interactions, thus spatially isolating MAVS monomer molecules from each other on the outer mitochondrial membrane and preventing their spontaneous aggregation (Qi et al., 2017). In addition to the main ORF1 and ORF2, three short upstream open reading frames (uORFs) exist in the 5′ untranslated mRNA regions (5′UTR) of the MAVS gene transcript mRNA. The uORF can reduce the translation initiation efficiency of downstream genes through various regulatory mechanisms and cause ribosome delay or trigger mRNA degradation, thereby inhibiting protein expression (Xu et al., 2017). Our group demonstrated that uORFs are *cis*-acting elements of MAVS transcripts that repress the downstream ORF translation by the leaky ribosomal scanning mechanism, limiting MAVS expression and preventing its spontaneous aggregation (Shi et al., 2020).

On the other hand, host cells maintain innate homeostasis by degrading protein aggregates, damaged organelles, or intracellular pathogens through autophagy. Mitophagy is a typical selective autophagy process and is mediated when the mitochondrial kinase PINK1 and E3 ubiquitin ligase Parkin promote mitochondrial outer membrane proteins’ ubiquitination (Lazarou et al., 2015). The formation of MAVS aggregates can increase intracellular ROS (Zhao et al., 2012). After recognizing this signal in resting cells, the mitochondrial protein Nix can directly bind to the ATG8 family member on the phagocytic protein to mediate PINK-PARKIN mitophagy and clear MAVS aggregates (Randow and Youle, 2014). Our group further discovered that in the absence of viral infection, endogenous MAVS produced by internal ORF-deleted transcripts could spontaneously aggregate and activate the IFN signaling pathway (Qi et al., 2017). Nix-mediated selective autophagy is initiated to prevent abnormal protein aggregation and maintain natural immune homeostasis (Shi et al., 2020).

## Conclusion and Future Perspectives

As one of the important signaling adaptors, MAVS has attracted extensive attention from researchers for its critical role in the antiviral signaling pathway. MAVS acts as an adaptor in RNA-sensing signaling pathways to induce IFN production by forming prion-like aggregates after receiving upstream signals from PRRs. This linking role and status determine that MAVS is an indispensable ingredient. After viral infection, MAVS regulates the immune response and inflammation levels in the cells through PTMs, such as phosphorylation and ubiquitination. Compelling evidence has shown that viruses have developed various strategies to evade the host’s innate immune surveillance by targeting MAVS. This phenomenon may be a crisis signal, or it may be a chance for a new field. The mitochondria where MAVS is localized are pivotal for energy metabolism and have emerged as vital platforms for MAVS-mediated antiviral signaling. O-linked β-N-acetylglucosamine mentioned in the previous article has proved that glucose metabolism has the MAVS regulation function (Li et al., 2018). Mitochondrial fission factor (Mff) can provide energy for MAVS disorganization. Acute antiviral immunity is mediated by Mff, which senses mitochondrial energy status and regulates the disorganization of MAVS clusters (Hanada et al., 2020). Still, the current research is mostly focused on the regulation of MAVS by mitochondrial dynamics. Whether the mitochondrial metabolic process might contribute to MAVS regulation in host-virus crosstalk is largely unknown and imperative to be elucidated. In summary, studying the mechanisms by which viruses counteract the MAVS-mediated immune response may provide new insights for the therapeutic strategy of viral infections.

As we know, the cGAS-STING pathway plays an important role in anti-tumor immunity. Targeting cGAS-STING has shown great progress in cancer therapy. Researches on the relevance between the RIG-I-MAVS signaling pathway in tumorigenesis and development are also in full swing. Recent reports have shown a significant reduction of MAVS expression in several human cancers and demonstrate its potential anti-tumor prospect (Hou et al., 2014). MAVS interacts with the tumor suppressor P53, maintaining the stability of P53 by inhibiting its ubiquitination and the formation of its P53-MDM2 complex, thereby inhibiting the development of tumors (Zhang et al., 2020a). Otherwise, epigenetic therapy is a novel cancer treatment method. This method can induce virus infection phenomenon to activate the MAVS signaling pathway and target cancer-initiating cells, resulting in a “viral mimicry” state with anti-tumor effects (Roulois et al., 2015). This work indicated that more roles of MAVS in tumorigenesis would be needed to reveal in future work.

Accidental and continuous activation of the immune system may lead to chronic inflammation and even autoimmune diseases. In recent years, the self-inhibition mechanism of how the immune system prevents the spontaneous aggregation of MAVS has gradually been clarified. Our knowledge of the regulatory mechanisms of immune homeostasis will remarkably improve our understanding of the immune system and provide new clues to the pathogenesis of chronic inflammation, autoimmune diseases, and cancer. Although the current investigations on the regulation of MAVS immune homeostasis are still limited, the advance of the field will undoubtedly benefit innate immunity studies. Based on these breakthroughs, this field is still worthy of further investigation.

## Author Contributions

NQ and YC conceived and wrote the first draft of the manuscript. YS provided substantial comments on the manuscript that were incorporated. JW depicted the illustrations. All authors contributed to the article and approved the submitted version.

## Funding

This work was supported by grants from the National Key Research and Development Program of China (2018YFA0900404), National Natural Science Foundation of China (31870864), Fundamental Research Funds for the Provincial Universities of Zhejiang (RF-B2020003), and Zhejiang Provincial Natural Science Foundation of China (LY20C080003). All sources of funding received for the research being submitted.

## Conflict of Interest

The authors declare that the research was conducted in the absence of any commercial or financial relationships that could be construed as a potential conflict of interest.

## Publisher’s Note

All claims expressed in this article are solely those of the authors and do not necessarily represent those of their affiliated organizations, or those of the publisher, the editors and the reviewers. Any product that may be evaluated in this article, or claim that may be made by its manufacturer, is not guaranteed or endorsed by the publisher.
